# Dysbiosis of lower respiratory tract microbiome are associated with proinflammatory states in non‐small cell lung cancer patients

**DOI:** 10.1111/1759-7714.15166

**Published:** 2023-12-02

**Authors:** Yangqian Li, Guanhua Rao, Guonian Zhu, Cheng Cheng, Lijuan Yuan, Chengpin Li, Jianpeng Gao, Jun Tang, Zhoufeng Wang, Weimin Li

**Affiliations:** ^1^ Department of Respiratory and Critical Care Medicine, Institute of Respiratory Health, Frontiers Science Center for Disease‐related Molecular Network, West China Hospital Sichuan University Chengdu China; ^2^ Genskey Medical Technology Co., Ltd Beijing China

**Keywords:** cytokine, dysbiosis, lung cancer, metagenomic sequencing, microbiome

## Abstract

**Background:**

The lung has a sophisticated microbiome, and respiratory illnesses are greatly influenced by the lung microbiota. Despite the fact that numerous studies have shown that lung cancer patients have a dysbiosis as compared to healthy people, more research is needed to explore the association between the microbiota dysbiosis and immune profile within the tumor microenvironment (TME).

**Methods:**

In this study, we performed metagenomic sequencing of tumor and normal tissues from 61 non‐small cell lung cancer (NSCLC) patients and six patients with other lung diseases. In order to characterize the impact of the microbes in TME, the cytokine concentrations of 24 lung tumor and normal tissues were detected using a multiple cytokine panel.

**Results:**

Our results showed that tumors had lower microbiota diversity than the paired normal tissues, and the microbiota of NSCLC was enriched in *Proteobacteria*, *Firmicutes*, and *Actinobacteria*. In addition, proinflammatory cytokines such as IL‐8, MIF, TNF‐ α, and so on, were significantly upregulated in tumor tissues.

**Conclusion:**

We discovered a subset of bacteria linked to host inflammatory signaling pathways and, more precisely, to particular immune cells. We determined that lower airway microbiome dysbiosis may be linked to the disruption of the equilibrium of the immune system causing lung inflammation. The spread of lung cancer may be linked to specific bacteria.

## Graphical abstract

The lung microbiome plays a crucial role in respiratory health, and its dysbiosis has been associated with lung cancer. This study investigated the relationship between microbiota dysbiosis and immune profiles within the tumor microenvironment (TME) in non‐small cell lung cancer (NSCLC) patients. Metagenomic sequencing was performed on tumor and normal tissues from 61 NSCLC patients and six patients with other lung diseases. Cytokine concentrations were measured in 24 lung tumor and normal tissues using a multiple cytokine panel. The results showed lower microbiota diversity in tumors compared to paired normal tissues, with enrichment of Proteobacteria, Firmicutes, and Actinobacteria in NSCLC. Proinflammatory cytokines, including IL‐8, MIF, and TNF‐α, were significantly upregulated in tumor tissues. Specific bacteria were linked to host inflammatory signaling pathways and immune cells. Dysbiosis in the lower airway microbiome may disrupt the equilibrium of the immune system and contribute to lung inflammation and the spread of lung cancer.

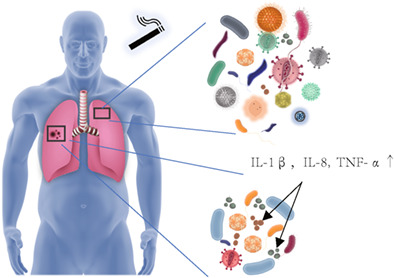

## INTRODUCTION

Lung cancer is the primary cause of cancer‐related deaths worldwide. In general, non‐small cell lung cancer (NSCLC) make up approximately 85% of lung cancers, while small cell lung cancer (SCLC) comprises 15% of lung cancer cases.^1^ From a histological point of view, lung adenocarcinoma (LUAD) and lung squamous cell carcinoma (LUSC) are the two major subtypes of NSCLC. While next‐generation sequencing technology has facilitated the identification of specific somatic mutations in NSCLC, these mutations are only present in approximately 30% of cases and have led to improved survival outcomes through targeted inhibition therapies.^2^ Recent studies have highlighted dysbiosis of the lower respiratory tract microbiome affects the progression of lung cancer, and microbiota emerging as crucial modulators in the carcinogenesis process and the immune response regulation against cancer cells.^3^ Several studies have demonstrated a significant abundance of *Granulicatella*, *Abiotrophia*, and *Streptococcus* at genus level, along with reduced community diversity in lung cancer patient samples compared to control samples.^4^ Our team has also observed that the lower respiratory tracts of lung cancer patients had less diversity in their microbiome.^5^ However, the specific profile and functional role of microbiota in tumor colonization among NSCLC patients remain largely unknown.

Lung cancer formation is tightly linked to chronic inflammation, which is defined by inflammatory cells infiltration and buildup of proinflammatory factors, including cytokines, which promote angiogenesis, cell proliferation, and tissue remodeling.^6^ An elevated risk of microbiota infection exists in lung cancer patients,^7^ and repeated exposure to microbiota alters the lung’ immune system.^8^ The commensal microbiota, a diverse group of bacteria that colonize the lung following exposure to the external environment, has been found to influence the efficacy of immunotherapeutic treatments for human tumors.^9^ Depending on the specific tumor microenvironment (TME), the microbiota may collaboratively modulate tumor‐promoting inflammation and antitumor immunity. Therefore, it is crucial to unravel the signaling pathways involved in the interactions between microbiota and host cells in the TME of NSCLC. Nevertheless, the precise signaling pathways involving microbiota and host cells in the TME of NSCLC are unknown.

In conclusion, the dysbiosis of the lower respiratory tract microbiome and its interactions with the host immune system play significant roles in NSCLC progression. Investigating the specific microbiota profiles, functional roles, and signaling pathways involved in the NSCLC TME will contribute to our understanding of tumor development and guide the development of targeted therapeutic approaches in the future.

In this study, we addressed this question using a combination of metagenomic sequencing of the microbiota in tumor and normal tissues from 61 NSCLC patients and six patients with other pulmonary diseases as control. As part of our investigation into the relationships between lung microbiota characteristics and clinical traits, as well as the host immune responses associated with microbiota of NSCLC patients, we also detected cytokines in the tumor tissues and their corresponding normal tissues from 24 NSCLC patients. These findings will offer a fresh look at the characteristics of the lung microbiota in NSCLC patients.

## METHODS

### Patients and clinical data collection

The enrollment for this study of patients who were diagnosed with NSCLC took place at West China Hospital of Sichuan University in China from 2020 to 2021. All patients included in the study underwent surgical treatment without receiving neoadjuvant therapy before surgery. Tumor samples and matched distal normal lung tissues located 5 cm away from the tumor margin were collected during the surgical procedure. Two pathologists examined each sample in order to determine the pathological diagnosis and tumor cellularity. The eighth edition of the American Joint Committee on Cancer's TNM method was used for cancer staging.

### Sample collection

Clinical sample collection followed the principles stated in the Helsinki declaration and was approved by the Institutional Review Board of West China Hospital of Sichuan University, China. Informed consent was obtained from the patients. Tumor samples and matched distal normal lung tissues (distant from the tumor 5 cm) were collected during surgery. Within 20 min of surgery, we first minced tumor tissues into tiny cubes <0.5 mm^3^, and then divided the tissues into two groups. One was frozen with liquid nitrogen for metagenomic sequencing, and the other was used for cytokine detection. The only equipment and supplies that came into contact with the lung tissues were sterile. In addition, we introduced six negative controls from sample collection, extraction and sequencing. The patients underwent surgical treatment, and neoadjuvant therapy was not administered before the surgical procedure. The Institutional Review Board of West China Hospital of Sichuan University gave its approval to this study (Chengdu, China; project identification code: 2020232).

### Genomic DNA extraction and metagenomic sequencing

Frozen tissue the size of a grain of rice and 500 μl PBS were first placed in a tissue lysis tube, then homogenized and lysed using a bead mill. Sample releasing agent (2007, Genskey) was used to remove hosts, and in accordance with the manufacturer's instructions, bacterial DNA was isolated using a microsample genomic DNA extraction kit (1901, Genskey). Using an NGS library construction kit (2014B, Genskey), the DNA libraries were created by performing DNA enzyme digestion (250–300 bp), end‐repair, A‐tailing, adapter ligation, and PCR amplification. An Agilent 2100 Bioanalyzer (Agilent Technologies) was used in conjunction with qPCR to measure the adapters prior to sequencing to evaluate the quality of the DNA libraries. Following a quality check, Illumina PE150 sequencing was carried out by combining several libraries according to the demands of effective concentration and target data amount. The sequencing tool utilized was the Illumina Novaseq 6000 (Illumina).

### Cell‐type composition analysis

In order to assess whether cell‐type compositions were significantly changed between different diseases, the percentages of each cell‐type in each sample were calculated and visualized as boxplot by R package ggpubr (version 0.4.0). We applied the Wilcoxon rank sum test to compare two independent groups and performed Kruskal–Wallis test to compare three or more independent groups. *p*‐values <0.05 were regarded as statistically significant.

### Microbiota analysis

Fastp (version 0.19.5) in particular,^10^ used to quality‐filter raw reads from samples, was used to check the data for adaptor contamination, low‐quality reads, and reads with low complexity. Using bowtie2 (version 2.3.4.3),^11^ reads that corresponded to the nucleotide sequence database (NT) and the human reference assembly GRCh38 were eliminated. Next, based on the NT (version 20 210 619), the mapped reads were classified with Kraken (version 2.1.2).^12^ After the above filtering, we retained the results in the annotated species (bacteria, fungi, and viruses) with a Perl script, normalizing the abundance to a data volume of 20 000 000 for subsequent analysis. Both alpha‐ and beta‐diversity analyses utilized QIIME2^13^ and R. Evaluating the differences in alpha‐diversity indices by the Wilcoxon rank‐sum test or Kruskal–Wallis of R package ggpubr (https://github.com/kassambara/ggpubr); three dimensionality reduction methods (nonmetric multidimensional scaling [NMDS], principal coordinate analysis [PcoA], and principal component analysis [PCA]) were used in beta‐diversity to observe the sample distribution. Difference analysis is based on relative abundance, using the Wilcoxon test to find out the species with differences between groups (*p* < 0.05, |FC| ≥ 10). We prefiltered out species with less than three detections within the group before differential analysis. We excluded the shared 392 species between six negative controls and the lung tissues to avoid being contaminated. Since tumor sizes, TNM stages, and smoking years have a greater impact on the tumor microecology, we also divided these three factors into different groups, and performed the above analysis under each group.

### Cytokine analysis

We used the Spearman rank correlation analysis and Fisher's exact test to evaluate the correlation between the cytokines and the different species under each group (*p* < 0.05, |R| ≥ 0.5). In addition, the cytokines under each group were analyzed with Wilcoxon rank‐sum test, and *p* < 0.05 was used as the filter condition to find the cytokines with differences between groups, and combined with the correlation results to infer the relationship between species and cytokines.

### Statistical analysis

*p*‐values <0.05 were regarded as statistically significant for all analyses, which were carried out using R software (version 4.0.3). The Wilcoxon rank‐sum test or the Kruskal–Wallis test was used to evaluate continuous variables. For beta‐diversity, statistical comparisons of weighted/unweighted UniFrac distances were performed by ANOSIM using the vegan package of the R software.

## RESULTS

### The overall profiles of lung microbiome

**FIGURE 1:**
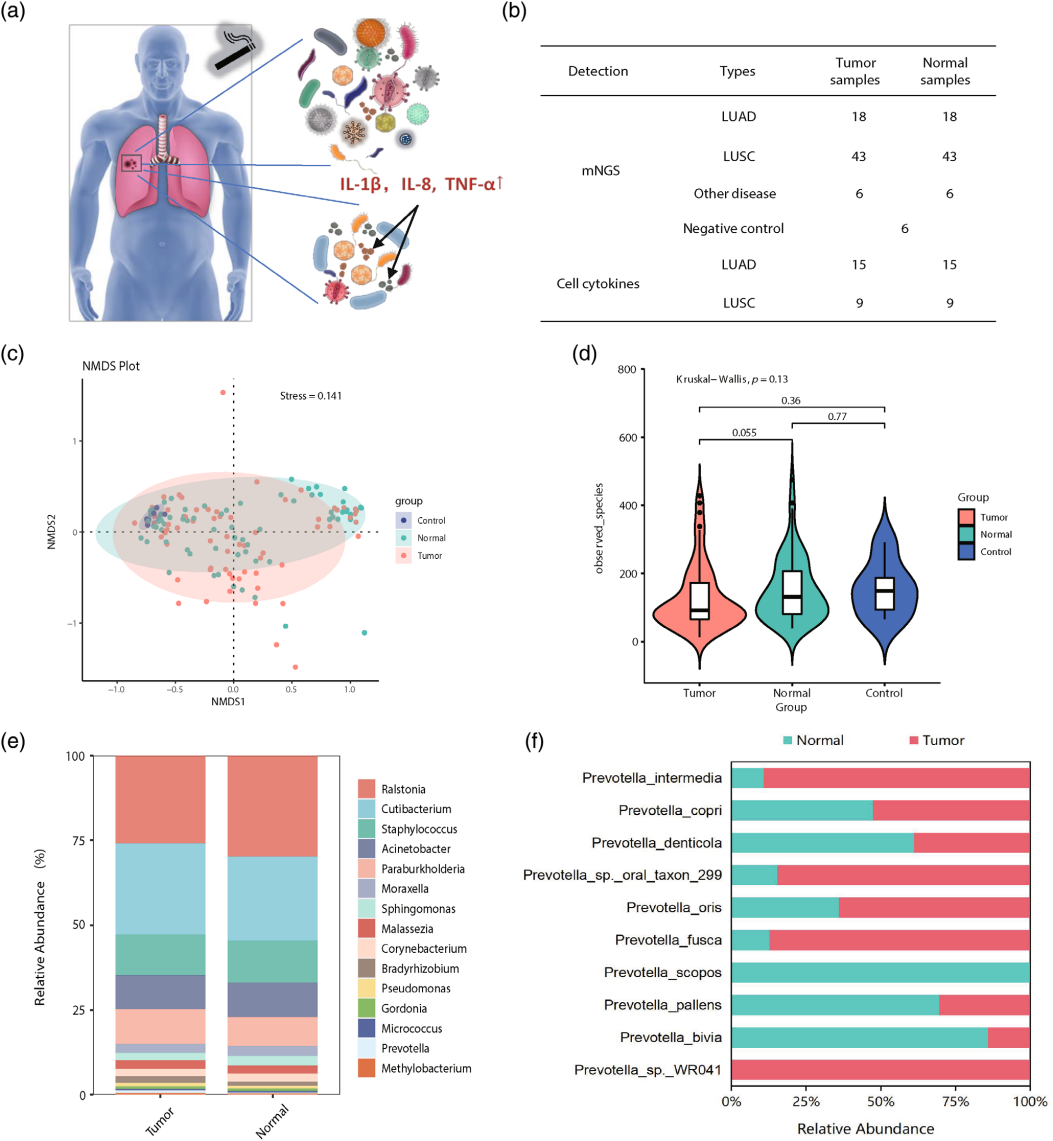
The overall lung microbiome profile. (a) Schematic diagram showing the microbiota and cytokines in lung tissues. (b) The number of human lung tissues and negative control analyzed in this study. (c) Nonmetric multidimensional scaling (NMDS) plot of tumors, normal tissues and negative controls shown in β‐diversity of microbiota. (d) The richness indices of observed species in three groups (Kruskal–Wallis, *p* = 0.13). (e) Composition of bacterial community and relative abundance at the genus level in tumor and normal tissues. (f) The relative abundance of *Prevotella* species between tumor and normal groups. LUAD, lung adenocarcinoma. LUSC, lung squamous cell carcinoma.

To characterize and visualize human NSCLC microbiota based on lung tissues, we performed metagenomic sequencing on 61 tumor tissues (tumor, *n* = 61) and their paired normal tissues (normal, *n* = 61), comprising 43 LUAD and 18 LUSC (Figure 1a, Tables S1 and S2). Beyond that, another six lung tissues of lung diseases were also sequenced. To address operating room or laboratory contaminants, environmental samples from collection and extraction were introduced as negative controls (Figure 1b), and we found the microbiota load was lower in negative control samples (Table S2). Among tissues, the cytokine concentrations of 15 LUAD patients and 9 LUSC patients within the same batch were detected using a 48‐cytokine panel (Figure 1b and Table S3). In addition, the Clinical Biochemistry‐Based Indexes (CBBI) of NSCLC patients were also included from West China Hospital, Sichuan University (Table S1).

To elucidate the microbiota community between tumor, normal tissues and negative controls, we used NMDS dimensionality reduction to compare the microbiota composition, and the results revealed a good confidence interval ellipse (stress <0.2) (Figure 1c). However, there was no significant difference in microbiota communities between tumor and normal tissues according to the observed species (Figure 1d). In general, we identified a total of 2034 microbes from 13 genera and four phyla in human lung tissues, and observed a noticeable trend of microbial alpha‐diversity that diminished from normal tissues to tumors. The prevalence of microbiota composition shown a difference between the two groups (Figure 1e). The relative proportions in the genera *Pseudomonas* and *Prevotella* accounted for 0.0369% and 0.0202% of the tumor tissues, respectively, while *Pseudomonas* represented 0.0265% in normal tissues, and *Prevotella* accounted for 0.0066% in normal tissues (Table 1). In particular, Prevotella, previously reported to be enriched in a proinflammatory condition,^14^, ^15^, ^16^, ^17^, ^18^ is highly enriched in tumor tissues (Figure 1f). On the other hand, clinically common pathogens have also been detected in tumor tissues (Table S4). For example, Klebsiella pneumoniae abounded in tumors (*p* < 0.05). Klebsiella pneumoniae is a bacterium that lives inside human intestines and can lead to pneumonia and infection if it gets into the lungs.^19^

**TABLE 1:** Proportion test of bacteria genera prevalence between tumor and normal tissues.

Genus	Specie count	Specie count percent	Normal relative abundance	All tumor relative abundance	Normal %	All tumor %
*Pseudomonas*	102	5%	0.006746555	0.009020868	0.026560006	0.036955841
*Corynebacterium*	56	3%	0.021033228	0.018579816	0.082804136	0.076116033
*Acinetobacter*	47	2%	0.086118486	0.082577945	0.339033395	0.338297523
*Streptococcus*	37	2%	0.002621589	0.003290632	0.010320735	0.013480751
*Microbacterium*	32	2%	0.000752492	0.000644785	0.002962429	0.002641496
*Polynucleobacter*	24	1%	0.001092721	0.00143483	0.004301851	0.005878074
*Staphylococcus*	23	1%	0.108939282	0.102694057	0.42887487	0.420707306
*Mycolicibacterium*	22	1%	0.000377318	0.00028507	0.001485436	0.00116785
*Chryseobacterium*	21	1%	0.000752727	0.000717358	0.002963355	0.002938806
*Sphingomonas*	20	1%	0.022126302	0.017926705	0.087107374	0.073440431
*Flavobacterium*	20	1%	7.02E‐05	7.98E‐05	0.000276357	0.000326755
*Prevotella*	19	1%	0.001682535	0.004935763	0.006623847	0.020220367
*Brevundimonas*	18	1%	0.001698376	0.001910991	0.00668621	0.007828768

We also examined the microbiota composition in LUAD, LUSC, and other lung diseases (Figure S1a, b). The results indicated that the relative abundance of the dominant microbiota was different in LUSC and LUAD, such as an enrichment of Acinetobacter in LUSC and Gordonia in LUAD. These findings may suggest that the microbiota in tumors is ecologically dysregulated compared to normal tissues.

### Inflammatory cytokine was significantly increased in tumors of NSCLC patients

**FIGURE 2:**
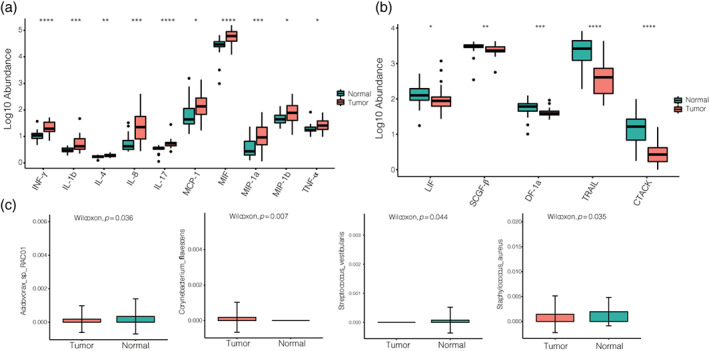
Concentrations of cytokines in tumor and normal tissues. (a) Box plots of 10 cytokines upregulated in tumor tissues and (b) five cytokines upregulated in normal tissues. Asterisks denote significance after testing correction as follows: *p‐value ≤ 0.05; **p‐value ≤ 0.01; ***p‐value ≤ 0.001; ****p‐value ≤ 0.0001. (c) Significant differences at species levels between tumor and normal tissues. (Wilcoxon, *p* < 0.05).

Lung is a dynamical balance and microbiota can interact with each other directly including mucous membranes, respiration and or indirectly via inflammatory cytokine. In this study, we aimed to assess the potential causal effect of lower airway dysbiosis to pulmonary immunity. We detected cytokine concentration between tumor and normal tissues. The results showed that 10 cytokines associated with proinflammatory including IL‐1β, IL‐4, IFN‐γ, MCP‐1, IL‐8, IL‐17, MIF, MIP‐1α, MIP‐1β, and TNF‐α were upregulated in tumors (Figure 2a), while the concentration of LIF, SCGF‐β, SDF‐1α, TRAIL, and CTACK were higher in normal tissues (Figure 2b).

To better understand the host–microbe interaction in lung cancer, we evaluated the association between the bacteria and lung tissues, and discovered that *Corynebacterium flavescens* was more prevalent in tumors (Figure 2c and Supporting Information Table S5). On the other hand, *Haemophilus influenzae*, *Staphylococcus aureus*, *Streptococcus vestibularis*, and *Acidovorax* sp. *RAC01* were found be more abundant in normal tissues (Figure 2c). This accords with the results that IL‐17 could respond to *Streptococcus agalactiae* and *Streptococcus salivarius* in NSCLC were remarkably elevated.^20^ We speculate that the inflammation caused by microbes may be the indirect promotion of proinflammatory cytokines.

### Microbiota signatures associated with stage in LUAD

**FIGURE 3:**
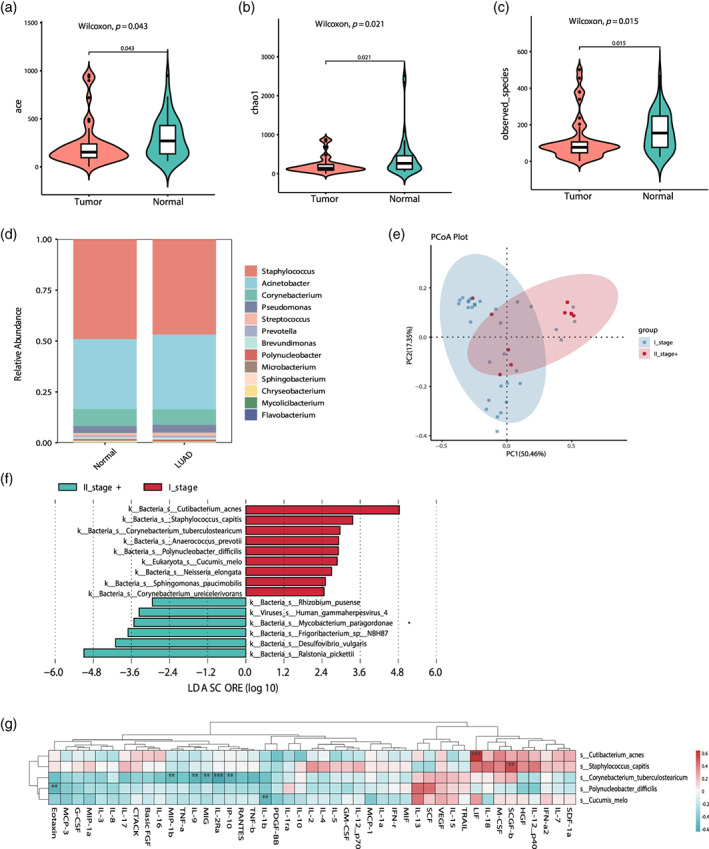
The microbiota diversity and correlation analysis of lung adenocarcinoma (LUAD) patients. (a–c) Alpha diversity between tumor and normal tissues were evaluated by (a) ACE, (b) Chao1 and (c) the observed species. (d) The relative abundance of bacterial community in LUAD patients at genus levels. (e) Principal coordinate analysis (PCoA) plots showed the microbiota beta diversity by clinical stage I and II–III groups in LUAD. (f) Differentially abundant species between LUAD in the stage I and II–III groups identified by linear discriminant analysis effect size (LEfSe). (g) Correlation analysis between different species of microbiota and cytokines in different groups.

In order to determine the microbiome variation between different types of lung tumors, we first estimated microbiome community richness and diversity within the LUAD samples. The alpha‐diversity index, including ACE and Chao1 were analyzed in tumor and normal tissues (Figure 3a–c). The results showed that the number of OTUs was significantly lower in tumors compared with normal tissues, and the diversity of the microbiota was also higher in normal groups. The dominant genera were abundant in *Streptococcus*, *Pseudomonas*, *Corynebacterium*, *Acinetobacter*, and *Microbacterium* in tumor and normal tissues (Figure 3d). We then evaluated microbiota differences based on the clinical LUAD stages, grouped as I (early stage) and II–III (later stage) stage of TNM classification. The PCoA and ANOSIM test showed that these two groups had a significant difference (Figure 3e, ANOSIM, *r* = −0.0555, *p* = 0.763). There was a significant enrichment of common pathogenic microbiota such as *Mycobacterium paragordonae*, *Desulfovibrio vulgaris*, and *human gammaherpesvirus*‐4 in stages II–III (Figure 3f). It was noteworthy that *Desulfovibrio* is commonly found in the human gut and is associated with intestinal diseases.^21^, ^22^, ^23^, ^24^ This may suggest “gut‐lung axis” existed between gut and lung.^25^, ^26^, ^27^, ^28^ Furthermore, *Cutibacterium acnes*, *Staphylococcus capitis*, and *Corynebacterium tuberculostearicum*, were enriched in stage I and correlated with certain cytokines (Figure 3g). In our study, its enrichment in stage I may have also played a beneficial role in the immune regulation of LUAD. The results of cytokine association analysis determined that *C. tuberculostearicum* was significantly and negatively correlated with MIP‐1b, IL‐9, MIG, IL‐2RA, and IP‐10 (Figure 3g). These findings further suggest that microbially triggered host inflammatory signaling pathways or microecological imbalances may play an important role in tumor development.

### Smoking inducing microbiota colonized in LUSC

**FIGURE 4:**
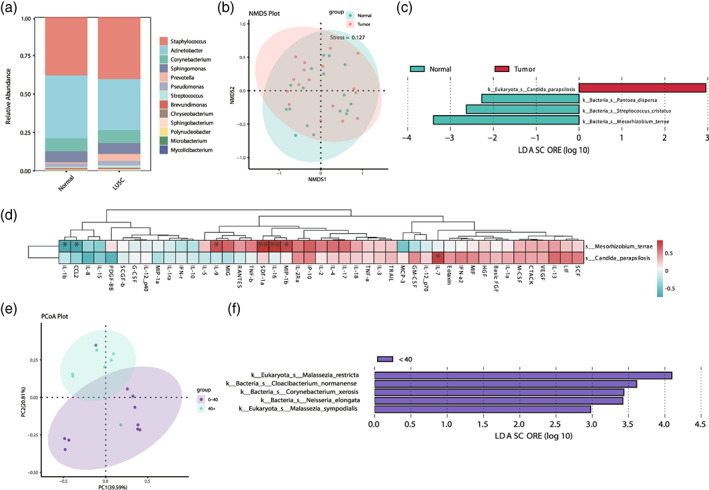
The microbiota diversity and correlation analysis of lung squamous cell carcinoma (LUSC) patients. (a) Stacked bar plot showing the bacteria relative abundance in LUSC tumors and normal tissues at genus levels. (b) The beta‐diversity analysis between normal and tumor from LUSC patients. (c) Species of microbiota with significant differences between LUSC and their normal tissues (|LDA| ≥ 2, *p* < 0.05). (d) Principal coordinate analysis (PCoA) plot of the microbiota in LUSC tumors size groups at species level. (e) The linear discriminant analysis effect size (LEfSe) identified the taxa with the greatest differences in abundance between <40 pack‐years LUSC patients and >40 pack‐years (|LDA| ≥ 2, *p* < 0.05). (f) Correlation analysis between different species of microbiota and cytokines in tobacco exposure histories (0–40 vs. >40 pack‐years).

To further assess the distribution of microbiota in LUSC, we observed a significant abundance of Staphylococcus and Acinetobacter as the predominant genera in LUSC (Figure 4a). In contrast to LUAD, the distinction of *Acinetobacter* was lower abundance in tumor tissues. The NMDS analysis provided visual separation between the two types of tissues (Figure 4b). Linear discriminant analysis effect size (LEfSe) showed that *Candida parapsilosis* was significantly enriched in tumor tissues while *Pantoea dispersa*, *Streptococcus cristatus*, and *Mesorhizobium terrae* were enriched in normal tissues (Figure 4c). The *C. parapsilosis*, an opportunistic fungal pathogen, was the most prevalent systemic nosocomial infection. Some *Candida* species, closely associated with increased expression of proinflammatory host immunity, were the most common model to determine the host‐fungus interaction.^29^, ^30^, ^31^, ^32^ Several studies have found that fungus can shape host immunity and contribute to carcinogenesis such as esophageal, colorectal, and pancreatic cancer.^33^, ^34^, ^35^, ^36^ Furthermore, in cytokine correlation analysis, a positive correlation between IL‐7 and *C. parapsilosis* was found (Figure 4d). It is well‐known that IL‐7 plays a critical role in the proliferation of T and B cells.^37^ It does appear that IL‐7 could promote tumor cell proliferation in LC by regulating the BCL2 gene family and promoting cFOS and cJUN activity in NSCLC.^38^, ^39^ We then evaluated the microbiota on the basis of tobacco exposure histories (0–40 vs. >40 pack‐years). In terms of smoking, PCoA was plotted to evaluate the similarity in two groups (Figure 4e). The different species, *Malassezia restricta*, *Malassezia sympodialis*, *Cloacibacterium normanense*, *Corynebacterium xerosis*, and *Neisseria elongate*, were all concentrated in the 0–40 pack‐years group (Figure 4f). Collectively, our results suggested that it is possible that fungi are also involved in LUSC microbiome imbalances and are associated with immune inflammatory responses.

## DISCUSSION

In this study, we integrated metagenomic sequencing to characterize the microbiome based on NSCLC tissues and provide a comprehensive method by which nearly all microbiota can be accurately identified, without the need for sequence‐specific amplification.^40^ Previous studies on the microbiota in the airways have primarily relied on samples obtained from bronchoalveolar lavage (BAL), bronchoscopic brushing, or sputum,^3^, ^41^, ^42^, ^43^, ^44^, ^45^, ^46^ which might be contaminated by oral microbiota in the upper respiratory tract, which affects the analysis and judgment of lung microbes.^47^ What is more, lung cancer studies have focused on genus levels.^3^, ^14^, ^17^, ^20^, ^48^ In our study, we found five core genera; *Pseudomonas*, *Corynebacterium*, *Acinetobacter*, *Streptococcus*, and *Microbacterium*, with high relative abundance in lung tissues. However, *Prevotella* (*Bacteroidetes*), *Streptococcus* (*Firmicutes*), and *Veillonella* (*Firmicutes*) genera, which are frequently prevalent in the oral cavity, have repeatedly been discovered in great quantity in BAL studies.^17^ Together, these findings suggest that the lung microbiota possesses unique characteristics.

Although the diversity and relative richness of microbiota detected in all tumor and normal tissues were not significantly different, the relative proportion of *Pseudomonas* (0.0369%), *Prevotella* (0.0202%), and *Streptococcus* (0.0134%) increased significantly in tumors of NSCLC patients. This observation aligns with previous studies that have reported an enrichment of *Streptococcus* in tumor tissues, suggested that new insights into the interaction between lung cancer and microbes, come from the changes in lower respiratory microbes.^14^, ^17^, ^49^ Despite the status, 96% bacteria and 4% fungi in TCGA primary tumors suggested that bacteria predominate in the tumor microbiome, and the TME is polymicrobial.^32^, ^35^ In our study, *C. acnes* was enriched in stage I. Although *C. acnes* is primarily well‐known as a skin microorganism, it could also become an opportunistic pathogen in other organs such as intestine, mouth, lungs and stomach.^50^, ^51^, ^52^, ^53^ Short chain fatty acids (SCFAs), which are known metabolic byproducts of *C. acnes*, have been linked to immune system modulation and the emergence of allergic disease,^54^, ^55^ and could regulate lung immune functions by regulating T cells and dendritic cell (DC) activity.^56^ These studies showed *C. acnes* had a beneficial effect on the host.

The host immune cells acquire information directly from the microbiota and the concurrent regional cytokine response. This information exchange modifies inflammatory responses and subsequently alters immune responses.^57^, ^58^, ^59^ In our study, we observed 10 elevated cytokine levels in tumor tissues of NSCLC, with the majority being cytokines involved in proinflammatory and regulatory cytokines like IL‐8, IFN‐γ as well as IL‐17.^60^ Among these cytokines, LIF as a pleiotropic cytokine, can substantially facilitate tissue protection during bacterial pneumonia, which is involved in tissue homeostasis.^61^, ^62^ The proinflammatory cytokines of IL‐1β, IL‐17, MIF, and TNF‐α were significantly increased in tumor represented complex immunity dysregulation and possible molecular mechanisms due to dysbiosis of the microbiome.^63^, ^64^, ^65^, ^66^, ^67^, ^68^ IL2RA can influence the activity of Treg cells to assist in the regulation of immunological tolerance.^69^ Targeting and eliminating Treg cells through the use of CD25 antibodies have been recognized as a key mechanism for tumor inhibition and the elimination of immunosuppression.^70^ Overall, our results suggest that lower airway dysbiosis in lung cancer is associated with distinct cytokine profiles and microbial compositions. The upregulation of proinflammatory cytokines and the presence of specific bacterial species highlight the intricate interplay between the host immune response, microbiota, and tumor development in the context of lung cancer. These findings contribute to our understanding of the complex mechanisms underlying lung cancer pathogenesis and provide insights for potential therapeutic interventions targeting the host–microbe interaction.

Importantly, the immune system plays a crucial role in maintaining the important aspects of host‐microbe symbiosis.^71^ In future studies, efforts will be directed towards developing methods for performing multiplex analyses with sorted cells as this will enable the identification of the cellular sources of different cytokines and provide deeper insights into the immune response in the context of lung cancer.

However, a limitation of the study was that the sample size in the groups was small, and may have been underpowered to detect important differences among the groups. Future large‐scale animal model studies will be required to investigate the function of the lung microbiome in the emergence of lung cancer, so that the lung microbiome can be successfully used as cancer biomarkers or microbiota treatments in lung cancer patients.

In conclusion, we profiled metagenomic sequencing of tumor tissues from NSCLC patients, and researchers discovered lower airway microbiome dysbiosis, which may upset the equilibrium of the immune system and cause lung inflammation. Members of *Pseudomonas* and *Prevotella* played an important role in the composition of the tumor microbial community. We also found pathogenic microbiota such as *Mycobacterium paragordonae* and *Human gammaherpesvirus*‐4 in later stage of LUAD, may be associated with inflammation. *Candida parapsilosis* is also significantly enriched in tumors, suggesting proinflammatory states in heavy smoking LUSC. The observed association between the microbiome and lung cancer biology may indicate an influence of the microbiota on the development and progression of lung cancer.

## AUTHOR CONTRIBUTIONS

Z. Wang, G. Rao, L. Yuan, and J. Gao performed metagenomic sequencing and processed the data. L. Yuan, J. Gao Y, and C. Cheng performed bioinformatic analyses. J. Tang obtained patient consent and collected the samples. Y. Li assisted in participant selection, consent, clinical information, and procurement of tissues. G. Zhu performed tissue dissociations and cytokine detection. W. Li provided clinical insights. Z. Wang and Y. Li analyzed and interpreted the data. Z. Wang and Y. Li conceived the study and wrote the manuscript. All authors contributed to the article and approved the submitted version.

## CONFLICT OF INTEREST STATEMENT

Authors Guanhua Rao, Lijuan Yuan, and Jianpeng Gao are employed by Genskey Medical Technology Co., Ltd, Beijing, China. The remaining authors declare that the research was conducted in the absence of any commercial or financial relationships that could be construed as a potential conflict of interest.

## Supporting information


**FIGURE S1.** (a) Composition of bacterial community and relative abundance among LUSC, LUAD, and other pulmonary diseases. (b) Venn diagram illustrates the overlap at specie levels in three groups.

**TABLE S1.** The samples and clinical information of metagenomic sequencing included in the study.
**TABLE S2.** The sample information of metagenomic sequencing.
**TABLE S3.** The detection results of 48 cytokines.
**TABLE S4.** List of pathogens detected in this study.
**TABLE S5.** List of bacteria that were detected in all tumors.
