# Supplementary figures and images for: Dysbiosis of lower respiratory tract microbiome are associated with proinflammatory states in non‐small cell lung cancer patients
Source: Thorac Cancer. 2023 Dec 2;15(2):111–21. doi: 10.1111/1759-7714.15166 (PMC10788479; doi:10.1111/1759-7714.15166)

A

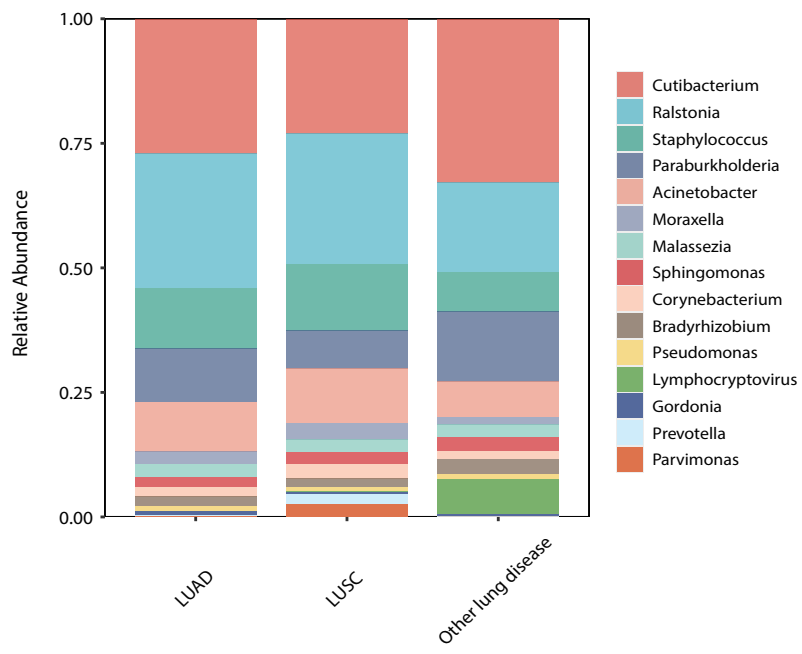

B

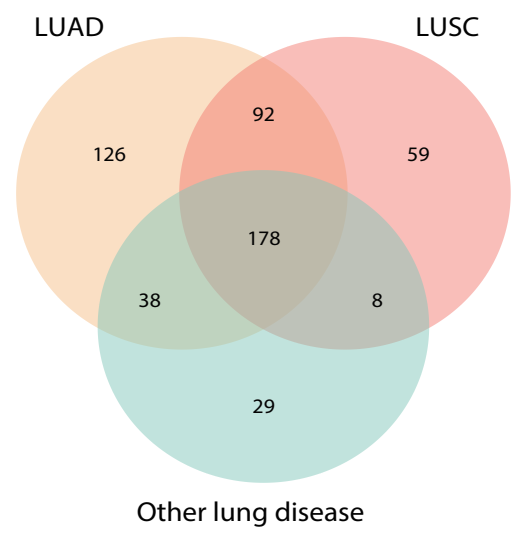

Supplement: Supplementary file 1 — FIGURE S1. (a) Composition of bacterial community and relative abundance among LUSC, LUAD, and other pulmonary diseases. (b) Venn diagram illustrates the overlap at specie levels in three groups. [file TCA-15-111-s002.pdf]
